# Key performance evaluation of commercialized multiplex rRT-PCR kits for respiratory viruses: implications for application and optimization

**DOI:** 10.1128/spectrum.01641-24

**Published:** 2024-10-29

**Authors:** Wanyu Feng, Yuqing Chen, Yanxi Han, Zhenli Diao, Zihong Zhao, Yuanfeng Zhang, Tao Huang, Yu Ma, Ziqiang Li, Jian Jiang, Jing Li, Jinming Li, Rui Zhang

**Affiliations:** 1 National Center for Clinical Laboratories, Institute of Geriatric Medicine, Chinese Academy of Medical Sciences, Beijing Hospital/National Center of Gerontology, Beijing, China; 2 National Center for Clinical Laboratories, Chinese Academy of Medical Sciences and Peking Union Medical College, Beijing, China; 3 Beijing Engineering Research Center of Laboratory Medicine, Beijing Hospital, Beijing, China; MultiCare Health System, Tacoma, Washington, USA

**Keywords:** multiple rRT-PCR, respiratory viruses, analytical sensitivity, competitive interference

## Abstract

Respiratory tract infections (RTIs) caused by viruses are prevalent and significant conditions in clinical settings. Accurate and effective detection is of paramount importance in the diagnosis, treatment, and prevention of viral RTIs. With technological advancements, multiplex real-time reverse transcription polymerase chain reaction (rRT-PCR) assays have been developed and extensively adopted for the diagnosis of viral RTIs. Given the potential challenges in the detection performance of multiplex assays, this study evaluated the analytical sensitivity and competitive interference of the six most commonly used multiplex rRT-PCR kits for detection of respiratory viruses in China. The results revealed that the limits of detection were variable across the viruses and kits. Most of the evaluated multiplex kits demonstrated comparable or enhanced analytical sensitivity compared with singleplex kits for clinically significant viruses, including human adenovirus (HAdV)-3, HAdV-7, Omicron BA.5, H1N1pdm09, H3N2, B/Victoria, respiratory syncytial virus subtype A, and respiratory syncytial virus subtype B, whereas multiplex kits showed relatively less analytical sensitivity for human rhinovirus-B72, human metapneumovirus-A2, parainfluenza virus (PIV)-1, and PIV-3. In addition, most multiplex kits successfully identified co-infections when one analyte was present at a low concentration and another analyte was present at a high concentration.

**IMPORTANCE:**

The complexity and severity of viral respiratory tract infections (RTIs) emphasize the pivotal role of precise diagnosis for viral RTIs in guiding effective public health responses and ensuring appropriate medical interventions, given the substantial population at risk. This study highlights the necessity and importance of evaluating the analytical validity of multiplex real-time reverse transcription polymerase chain reaction assays, offering valuable insights into their optimization and application.

## INTRODUCTION

Viral respiratory tract infection (RTI) is a major global health concern associated with high morbidity and mortality rates (1). Public health emergencies, including four pandemic influenza outbreaks in 1918, 1957, 1968, and 2009, severe acute respiratory syndrome (SARS) in 2002, Middle East respiratory syndrome in 2012, and coronavirus disease in 2019 (2–4), are responsible for growing concern about viral RTIs. A study conducted by the Chinese Centers for Disease Control and Prevention on acute RTIs in China from 2009 to 2019 revealed that influenza virus is the most commonly identified respiratory viruses, followed by respiratory syncytial virus (RSV), human rhinovirus (HRV), parainfluenza virus (PIV), human adenovirus (HAdV), human coronavirus, human bocavirus, and human metapneumovirus (hMPV) (3).

Accurate and effective diagnosis is vital for expediting the implementation of appropriate antiviral treatment and infection control measures for the management of viral RTIs; the detection methods should have high sensitivity and specificity (5, 6). Conventional detection methods, such as viral culture and immunofluorescence assays for respiratory viruses, are replaced by convenient and sensitive nucleic acid-based methods. Real-time reverse transcription polymerase chain reaction (rRT-PCR), which is distinguished by its specificity, sensitivity, and compatibility with full automation, is recognized as the “gold standard” for diagnosis of the viral RTIs (7, 8). Owing to the limited availability of fluorophores and the prevalent utilization of monochromatic energizing light sources, the initial generation of *in vitro* diagnostic medical devices for nucleic acid-based molecular tests primarily focused on the detection or quantitation of a singular nucleic acid sequence within a clinical specimen (9). Viral RTIs are characterized by overlapping epidemic peaks and similar clinical symptoms, which complicate differential diagnosis. The primary limitation of singleplex rRT-PCR assay is the identification of only one target, resulting in likely missing infections than that of multiplex rRT-PCR assay. Combination of multiple singleplex rRT-PCR assays to detect targets results in costs associated with samples, reagents, personnel, and time consumption, posing significant challenges in resource-limited emergencies (6). Advances in real-time PCR have spurred rapid evolution in both amplification hardware and fluorogenic detection chemistry, enabling the development of multiplex rRT-PCR assays tailored for the concurrent identification of numerous targets in a clinical sample.

Since introduction, multiplex rRT-PCR has been successfully applied in syndromic diagnosis for RTIs, particularly during the notable prevalence of influenza virus along with other respiratory viruses in circulation and in cases of co-infections in immunocompromised individuals and patients with critical illness (8, 10–16). However, the likelihood of interactions between multiple primer pairs and preferential amplification of one target sequence over another are known phenomena in multiplex PCR assays, both of which can compromise analytical sensitivity and introduce competitive interference (14, 17, 18). Reviews and research have delineated the clinical performance of available multiplex assays, in which multiplex rRT-PCR assays were performed with high diagnostic accuracy and provided an ideal method for identifying co-infection compared to reference methods when the virus was abundant in the clinical samples. However, most discordant results were observed when the viral load was low, suggesting differences in analytical sensitivity among methods, particularly between multiplex and singleplex rRT-PCR assays (10–12, 19–23). Reduced analytical sensitivity may have a noticeable impact on the detection of low-level viral shedding in populations comprising infants, young children, and adults using multiplex rRT-PCR assays (20, 24, 25). Competitive interference may fail to identify co-infections when an analyte is at a low load and other analytes are at a high load (26–31).

**Fig 1:**
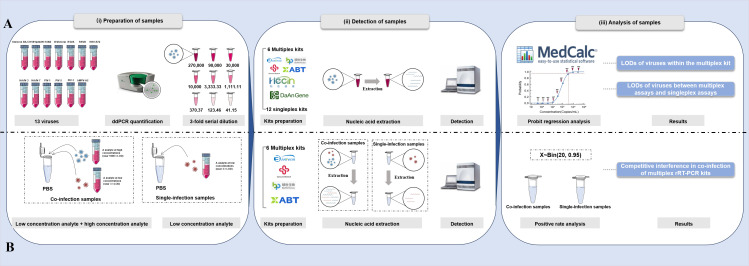
Schematic of the study design. (**A**) The evaluation of the analytical sensitivity of multiplex rRT-PCR kits and singleplex rRT-PCR kits for respiratory viruses. (**B**) The evaluation of competitive interference of multiplex rRT-PCR kits for respiratory viruses. Omicron BA.5, severe acute respiratory syndrome coronavirus 2 Omicron BA.5 strain; H1N1pdm09, pandemic influenza A (H1N1) 2009 virus; H3N2, influenza A (H3N2) virus; B/Victoria, Victoria lineage of influenza B virus; HAdV-3, -7, human adenovirus type 3, 7; RSVA, respiratory syncytial virus subtype A; RSVB, respiratory syncytial virus subtype B; PIV-1, -2, -3, parainfluenza virus types 1, 2, 3; hMPV-A2, human metapneumovirus A2 strain; HRV-B72, human rhinovirus B72 strain.

With the increasing availability and application of multiplex rRT-PCR assays for viral RTIs, there is a growing need for comprehensive performance evaluation to consistently achieve their intended utility (32, 33). Therefore, this study aimed to evaluate the analytical sensitivity and competitive interference of the six most commonly used multiplex rRT-PCR kits for respiratory viruses in China and to provide useful insights into the optimization and application of multiplex assays (Fig. 1).

## MATERIALS AND METHODS

### Preparation of commercial rRT-PCR kits

The main manufacturers of respiratory virus rRT-PCR kits in China include Sansure Biotech Co., Ltd. (Sansure), Beijing Applied Biological Technologies Co., Ltd. (ABT), Shanghai BioGerm Medical Technology Co., Ltd. (BioGerm), Jiangsu BioPerfectus Technologies Co., Ltd. (BioPerfectus), Guangzhou Hecin Health Co., Ltd. (Hecin), and Daan Gene Co., Ltd. of Sun Yat-Sen University (Daan). The six commercial multiplex rRT-PCR kits used for the evaluation included Six Respiratory Pathogens Nucleic Acid Diagnostic Kit (Sansure 6-plex), SARS-CoV-2 and Influenza A/B Virus Nucleic Acid Diagnostic Kit (Sansure 3-plex), Multiplex Real-Time PCR Diagnostic Kit for Rapid Detection of Six Respiratory Viruses (ABT 6-plex), Influenza A/B Viral Nucleic Acid Detection Kit (BioGerm 2-plex), 6-in-1 Respiratory Virus Nucleic Acid Diagnostic Kit (BioGerm 6-plex), and Influenza A and B Viruses Real-time PCR Kit (BioPerfectus 2-plex). Twelve matching singleplex rRT-PCR kits were used for comparison. The characteristics of these kits are shown in Table 1 and Table S3.

**TABLE 1:** Characteristics of selected commercially rRT-PCR kits for respiratory viruses^*a*^

Manufacturers	Detection kits	Targets and claimed LODs	Nucleic acid extraction reagent and instrument	Calculated LODs with 95% CI (copies/mL)
Sansure Biotech Co., Ltd. (Sansure)	Sansure 6-plex	Influenza A virus: 2.0 TCID_50_/mL Influenza B virus: 2.0 TCID_50_/mL RSV: 500 copies/mL HAdV: 500 copies/mL HRV: 500 copies/mL MP: 500 copies/mL	Sansure Nucleic Acid Extraction-Purification Kit (Cata No. S10025) Sansure Natch system (S-F11C) instrument	H1N1pdm09: 5226.31 (3624.79–10946.00) H3N2: 5253.32 (3519.40–11293.46) B/Victoria: 1152.08 (689.28–2861.37) RSVA: 23004.04 (16256.72–48275.18) RSVB: 23506.45 (15617.59–50856.18) HAdV-3: 865.29 (241.95–2268.54) HAdV-7: 4425.71 HRV-B72: 11257.39 (7751.92–23649.74)
	Sansure 3-plex	SARS-CoV-2: 200 copies/mL Influenza A virus: 200 copies/mL Influenza B virus: 200 copies/mL		Omicron BA.5: 266.79 (169.55–741.84) H1N1pdm09: 4128.51 (2544.73–9501.94) H3N2: 4598.84 (3079.58–9874.67) B/Victoria: 1128.08 (679.72–2769.21)
	Sansure singleplex	SARS-CoV-2: 200 copies/mL		Omicron BA.5: 332.49 (219.46–770.06)
	Sansure singleplex	Influenza A virus: 6.5 TCID_50_/mL		H1N1pdm09: 4530.73 (3260.06–8979.36) H3N2: 9447.72 (6662.24–19745.98)
	Sansure singleplex	HAdV: 200 copies/mL		HAdV-3: 423.94 (241.95–1485.77) HAdV-7: 3748.05 (2363.97–8453.87)
Beijing Applied Biological Technologies Co., Ltd. (ABT)	ABT 6-plex	Influenza A virus: 1 × 10^3^ copies/mL HAdV: 1 × 10^3^ copies/mL RSV: 1 × 10^3^ copies/mL PIV-3: 1 × 10^3^ copies/mL Influenza B virus: 1 × 10^3^ copies/mL PIV-1: 1 × 10^3^ copies/mL	ABT Nucleic Acid Extraction Kit (Cata No. CN8043(I)−64T) ABT MagiCube48 instrument	H1N1pdm09: 4319.41 (2871.65–9321.61) H3N2: 1475.42 (1080.71–2014.31) HAdV-3: 746.74 (455.94–1856.54) HAdV-7: 441.43 (318.77–874.07) RSVA: 1555.05 RSVB: 11662.53 PIV-3: 17841.01 (11113.20–40665.86) B/Victoria: 11523.04 (7981.17–24122.65) PIV-1: 6596.55 (4154.70–14894.73)
Shanghai BioGerm Medical Technology Co., Ltd. (BioGerm)	BioGerm 2-plex	Influenza A virus: 1 × 10^3^ copies/mL Influenza B virus: 1 × 10^3^ copies/mL	BioGerm Nucleic Acid Extraction Kit (Cata No. TQ-BG-008–96D) BioGerm BG-Abot-96 instrument	H1N1pdm09: 35887.75 (21915.94–83223.05) H3N2: 27096.39 (19086.24–56726.95) B/Victoria: 23506.45 (15617.59–50856.18)
	BioGerm 6-plex	PIV-1: 1 × 10^3^ copies/mL HAdV: 1 × 10^3^ copies/mL hMPV: 1 × 10^3^ copies/mL RSV: 1 × 10^3^ copies/mL PIV-2: 1 × 10^3^ copies/mL PIV-3: 1 × 10^3^ copies/mL		PIV-1: 15110.92 (9804.97–33278.53) HAdV-3: 325.06 (209.83–804.50) HAdV-7: 812.10 (507.90–1909.52) hMPV-A2: 11988.53 RSVA: 5111.24 RSVB: 28902.21 (18422.88–64605.87) PIV-2: 5698.09 (4144.48–11510.34) PIV-3: 11419.10 (7548.87–24751.89)
	BioGerm singleplex	SARS-CoV-2: 150 copies/mL		Omicron BA.5: 659.77 (444.77–1417.14)
Jiangsu BioPerfectus Technologies Co., Ltd. (BioPerfectus)	BioPerfectus 2-plex	Influenza A virus: 2.0 TCID_50_/mL Influenza B virus: 2.0 TCID_50_/mL	QIAamp Viral RNA Mini Kit (Cata No. 52904)	H1N1pdm09: 1405.20 H3N2: 4214.27 (1089.23–2438.32) B/Victoria: 4995.86
Daan Gene Co., Ltd. of Sun Yat-Sen University. (Daan)	Daan singleplex	SARS-CoV-2: 100 copies/mL	Daan nucleic acid isolation or purification reagent (Cata No. DA062X) Daan Smart 32 instrument	Omicron BA.5: 513.22 (353.23–1082.86)
	Daan singleplex	Influenza A virus: 1.0 × 10^3^ PFU/mL		H1N1pdm09: 9774.59 (6041.85–22427.12) H3N2: 8854.06 (5921.66–19032.50)
	Daan singleplex	Influenza B virus: 1.0 × 10^3^ PFU/mL		B/Victoria: 13366.57
	Daan singleplex	RSV: 1 × 10^3^ PFU/mL		RSVA: 27096.39 (19086.24–56726.95) RSVB: 23004.04 (16256.72–48275.18)
	Daan singleplex	HRV: 200 copies/mL		HRV-B72: 10618.78 (6908.46–23333.62)
	Daan singleplex	hMPV: 200 copies/mL		hMPV-A2: 1539.29 (1059.67–3241.09)
Guangzhou Hecin Health Co., Ltd. (Hecin)	Hecin singleplex	PIV-1: 0.03 TCID_50_/0.1 mL	Hecin PIV-1 Nucleic Acid Test Kit (PCR-fluorescence probe method)	PIV-1: 1840.73 (1310.95–3743.63)
	Hecin singleplex	PIV-3: 0.02 TCID_50_/0.1 mL	Hecin PIV-3 Nucleic Acid Test Kit (PCR-fluorescence probe method)	PIV-3: 1424.05 (1089.23–2438.32)

^*a*^ LODs, limit of detections; Omicron BA.5, severe acute respiratory syndrome coronavirus 2 Omicron BA.5 strain; H1N1pdm09, pandemic influenza A (H1N1) 2009 virus; H3N2, influenza A (H3N2) virus; B/Victoria, Victoria lineage of influenza B virus; HAdV-3, -7, human adenovirus type 3, 7; RSVA, respiratory syncytial virus subtype A; RSVB, respiratory syncytial virus subtype B; PIV-1, -2, -3, parainfluenza virus types 1, 2, 3; hMPV-A2, human metapneumovirus A2 strain; HRV-B72, human rhinovirus B72 strain; MP, *Mycoplasma pneumoniae*; TCID_50_, the 50% tissue culture infective dose; PFU, plaque-forming unit.

### Preparation of samples

Based on the pathogen spectrum of the selected kits and the epidemiological features of viral RTIs (3), we selected major subtypes of 13 viruses for this study. These included SARS-CoV-2 Omicron BA.5 strain (Omicron BA.5), pandemic influenza A (H1N1) 2009 virus (H1N1pdm09), influenza A (H3N2) virus (H3N2), Victoria lineage of influenza B virus (B/Victoria), RSV subtypes A and B (RSVA and RSVB), HRV-B72 strain, HAdV types 3 and 7 (HAdV-3 and HAdV-7), PIV types 1, 2, and 3 (PIV-1, PIV-2, and PIV-3), and hMPV-A2 strain. Owing to the challenges in acquiring comprehensive positive clinical specimens, inactivated cell culture supernatants of individual viruses purchased from Hecin Health Co., Ltd. (China) and Sinopharm Group Co., Ltd. (China) were used. These viruses were verified using real-time quantitative PCR (RT-qPCR; TaKaRa, Japan), after which they were quantified using droplet digital PCR (ddPCR; Bio-Rad, USA). Viral RNA and DNA were extracted using the QIAamp Viral RNA Mini Kit (Qiagen, Germany) and QIAamp Blood DNA Mini Kit (Qiagen, Germany), respectively. Extracted viral RNAs were reverse transcribed to cDNA using PrimeScript RT Reagent Kit (Perfect Real Time) (TaKaRa, Japan). ddPCR was performed on QX-200 system (Bio-Rad, USA) using Supermix for Probes (Bio-Rad, USA). All primer and probe sequences used for RT-qPCR and ddPCR were based on the published literature and are listed in Table S1. Recognizing the challenges associated with standardizing specimen preservation solutions and type of samples across various kits, we used phosphate-buffered saline (PBS; Viva Cell, China) dilutions of inactivated pure viral cultures (34). In addition, a simple matrix equivalency study was performed to establish the equivalency of dilution of inactivated cell cultured supernatants in a simulated clinical matrix with PBS. The representative result of the matrix equivalency study showed no statistically significant difference in the performance of multiplex kits when compared between the simulated clinical matrix and PBS (Table S2).

### Limits of detections of different kits for different strains

The quantified viral samples were serially diluted threefold to 270,000, 90,000, 30,000, 10,000, 3333.33, 1111.11, 370.37, 123.46, and 41.15 copies/mL using PBS. Positive samples with 20 replicates per concentration were tested using the selected kits on Applied Biosystems 7500 Fast Real-Time PCR System (Thermo Fisher Scientific, USA). All procedures, including nucleic acid extraction, detection, and interpretation of results, were performed according to the manufacturer’s instructions.

### Competitive interference assays of multiplex rRT-PCR kits

If one target in a respiratory viral panel multiplex assay is present at high levels, the detection of another target at low levels may be impaired (32, 33). To evaluate the internal competitive interference of the six selected multiplex kits, we used three replicates of simulated co-infection samples containing one analyte at low concentrations [approximately 3 × limit of detection (LOD)] and another at high concentrations (approximately 1,000 × LOD), according to the FDA guidance on the respiratory viral panel multiplex nucleic acid assay and the procedures outlined in FDA-approved multiplex rRT-PCR assays (26, 27, 32, 35). In addition, three replicates of simulated single-infection samples containing one target analyte at low concentrations (approximately 3 × LOD) were used as controls. In the event of any failure across the three replicates, a supplementary validation procedure with 20 additional replicates was conducted for both simulated co-infection and single-infection samples. Based on the binomial distribution with *N* = 20 and *P* = 0.95 [X ~ Bin(20, 0.95)], the identification of a competitive interference effect was defined as 16 or fewer positive results in co-infection samples (35).

### Statistical analysis

MedCalc Statistical Software version 19.6.1 (MedCalc Software Ltd., Ostend, Belgium) was used for probit regression analysis to estimate the LODs of the selected kits. SPSS Statistics for Windows (version 19.0; IBM Corp., Armonk, NY, USA) was used for independent sample *t*-tests to establish the equivalency of the matrix equivalency study. Statistical significance was set at *P* < 0.05.

## RESULTS

### Calculated LODs of the selected kits for different viruses

The calculated LODs with a 95% repeatability are presented in Table 1. The calculated LODs differed across different viruses and rRT-PCR kits; the LODs reflect the entire process of nucleic acid extraction and detection. Accordingly, the calculated LODs were further analyzed and compared based on the kit and virus types. The calculated LODs differed the most in case of H1N1pdm09, ranging from 1405.20 to 35887.75 copies/mL according to the kit type. Additionally, the calculated LODs differed the most for various targets in a multiplex assay, ranging from 325.06 to 28902.21 copies/mL.

### Comparing the calculated LODs between multiplex kits and singleplex kits

**Fig 2:**
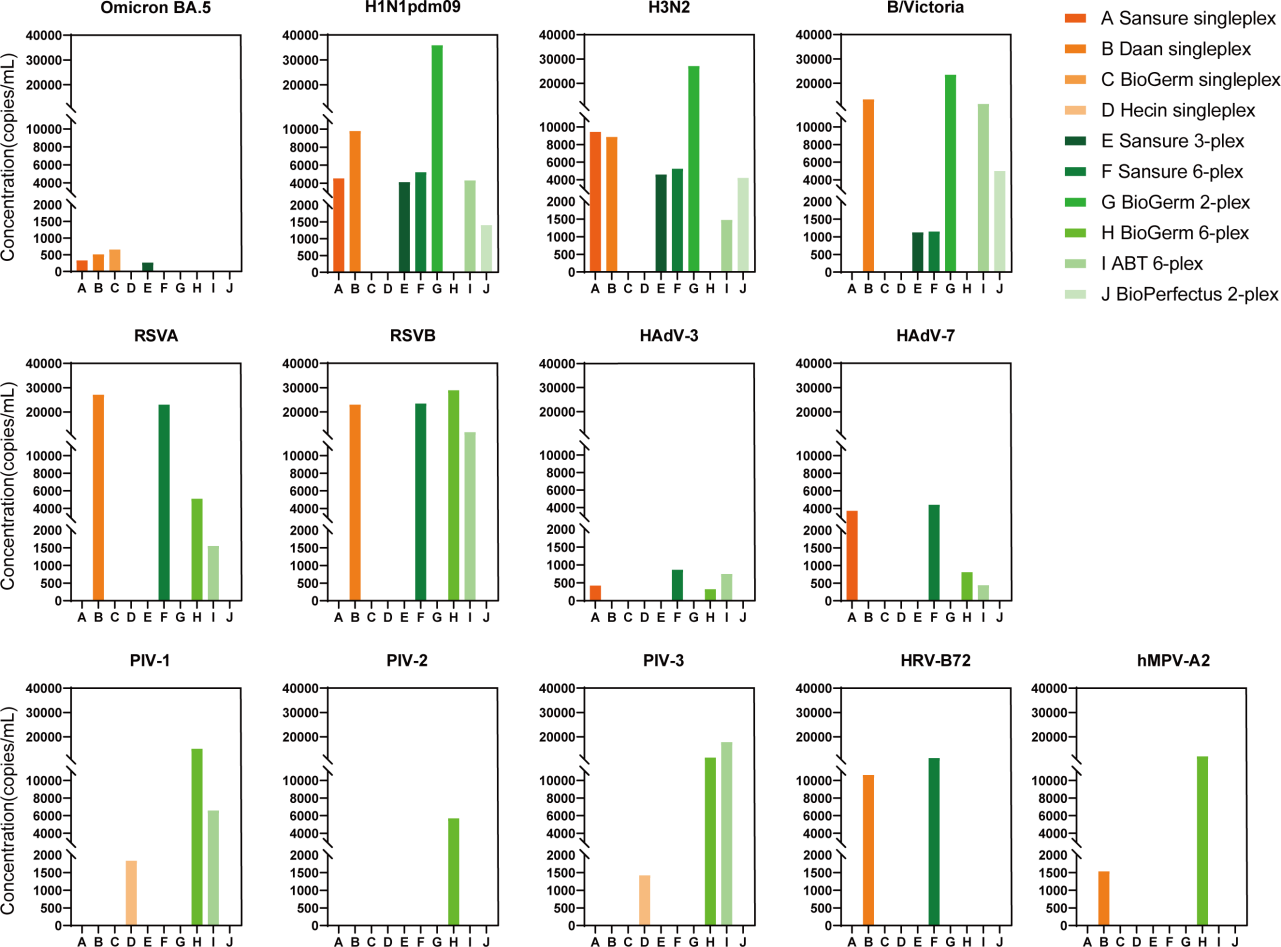
Calculated LODs for 13 viruses. Omicron BA.5, severe acute respiratory syndrome coronavirus 2 Omicron BA.5 strain; H1N1pdm09, pandemic influenza A (H1N1) 2009 virus; H3N2, influenza A (H3N2) virus; B/Victoria, Victoria lineage of influenza B virus; HAdV-3, -7, human adenovirus type 3, 7; RSVA, respiratory syncytial virus subtype A; RSVB, respiratory syncytial virus subtype B; PIV-1, -2, -3, parainfluenza virus types 1, 2, 3; hMPV-A2, human metapneumovirus A2 strain; HRV-B72, human rhinovirus B72 strain.

The comparison of the analytical sensitivities of multiplex and singleplex kits is shown in Table 1 and Fig. 2. The calculated LODs for the same virus differed among various kits. The majority of the selected multiplex rRT-PCR kits exhibited equivalent or improved analytical sensitivity compared with singleplex kits for the viruses HAdV-3, HAdV-7, Omicron BA.5, H1N1pdm09, H3N2, B/Victoria, RSVA, and RSVB. However, for HRV-B72, hMPV-A2, PIV-1, and PIV-3, multiplex kits demonstrated relatively less analytical sensitivity compared with singleplex kits.

### Comparing the calculated LODs for different viruses within the same multiplex kit

**Fig 3:**
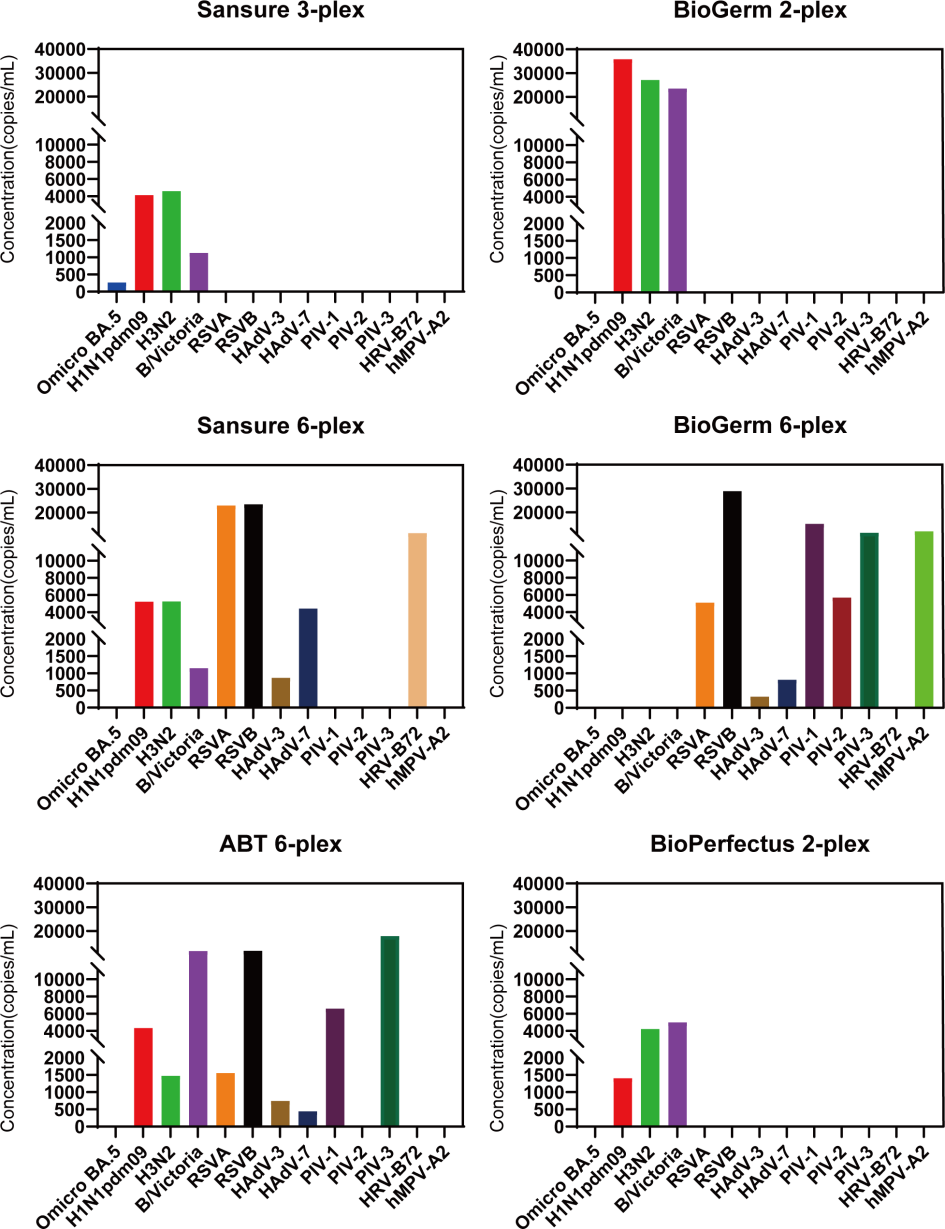
Calculated LODs for six multiplex kits. Omicron BA.5, severe acute respiratory syndrome coronavirus 2 Omicron BA.5 strain; H1N1pdm09, pandemic influenza A (H1N1) 2009 virus; H3N2, influenza A (H3N2) virus; B/Victoria, Victoria lineage of influenza B virus; HAdV-3, -7, human adenovirus type 3, 7; RSVA, respiratory syncytial virus subtype A; RSVB, respiratory syncytial virus subtype B; PIV-1, -2, -3, parainfluenza virus types 1, 2, 3; hMPV-A2, human metapneumovirus A2 strain; HRV-B72, human rhinovirus B72 strain.

The calculated LODs using the same multiplex kit varied across different targets (Table 1; Fig. 3). A multiplex rRT-PCR kit designed to detect two respiratory viruses typically includes influenza A and B viruses. In case of BioPerfectus 2-Plex Kit, the calculated LODs for H1N1pdm09 and H3N2 were lower than those for B/Victoria, whereas in case of BioGerm 2-Plex Kit, the calculated LOD for B/Victoria was lower. For the Sansure 3-Plex Kit, the calculated LOD for Omicron BA.5 was the lowest. The Sansure, BioGerm, and ABT 6-plex kits showed the maximum analytical sensitivity for HAdV-3, HAdV-3, and HAdV-7, respectively. The differences in the calculated LODs were reflected in varied positive rates at equivalent concentrations. However, at viral concentrations >30,000 copies/mL, all the selected kits demonstrated a 100% positivity rate (Table 2).

**TABLE 2:** Summary of positive rates for serially diluted virus samples^*a*^

Detection kit	Virus samples	Rate of no. detected/no. replicates (%) at each concentration (copies/mL)									
		270,000	90,000	30,000	10,000	3333.33	1111.11	370.37	123.46	41.16	0
Sansure singleplex	Omicron BA.5	100	100	100	100	100	100	95	75	10	0
Sansure singleplex	H1N1pdm09	100	100	100	100	85	15	0	0	0	0
	H3N2	100	100	100	100	30	5	0	0	0	0
Sansure singleplex	HAdV-3	100	100	100	100	100	100	95	65	40	0
	HAdV-7	100	100	100	100	100	40	30	0	0	0
BioGerm singleplex	Omicron BA.5	100	100	100	100	100	100	75	25	0	0
Daan singleplex	Omicron BA.5	100	100	100	100	100	100	85	30	0	0
Daan singleplex	H1N1pdm09	100	100	100	100	50	45	0	0	0	0
	H3N2	100	100	100	100	50	15	0	0	0	0
Daan singleplex	B/Victoria	100	100	100	50	0	0	0	0	0	0
Daan singleplex	RSVA	100	100	100	35	5	0	0	0	0	0
	RSVB	100	100	100	50	5	0	0	0	0	0
Daan singleplex	HRV-B72	100	100	100	100	40	20	0	0	0	0
Daan singleplex	hMPV-A2	100	100	100	100	100	85	30	0	0	0
Hecin singleplex	PIV-1	100	100	100	100	100	75	10	0	0	0
Hecin singleplex	PIV-3	100	100	100	100	100	85	5	0	0	0
Sansure 3-plex	Omicron BA.5	100	100	100	100	100	100	100	70	30	0
	H1N1pdm09	100	100	100	100	100	50	10	10	0	0
	H3N2	100	100	100	100	90	30	5	0	0	0
	B/Victoria	100	100	100	100	100	100	55	40	5	0
Sansure 6-plex	H1N1pdm09	100	100	100	100	80	20	0	0	0	0
	H3N2	100	100	100	100	80	35	0	0	0	0
	B/Victoria	100	100	100	100	100	100	60	30	10	0
	RSVA	100	100	100	50	5	0	0	0	0	0
	RSVB	100	100	100	60	20	0	0	0	0	0
	HRV-B72	100	100	100	90	45	0	0	0	0	0
	HAdV-3	100	100	100	100	100	100	75	40	15	0
	HAdV-7	100	100	100	100	50	0	0	0	0	0
BioGerm 2-plex	H1N1pdm09	100	100	100	45	30	5	0	0	0	0
	H3N2	100	100	100	35	5	0	0	0	0	0
	B/Victoria	100	100	100	60	20	0	0	0	0	0
BioGerm 6-plex	RSVA	100	100	100	100	10	0	0	0	0	0
	RSVB	100	100	100	50	30	0	0	0	0	0
	PIV-1	100	100	100	90	30	10	0	0	0	0
	PIV-2	100	100	100	100	70	5	0	0	0	0
	PIV-3	100	100	100	100	30	15	0	0	0	0
	HAdV-3	100	100	100	100	100	100	95	70	15	0
	HAdV-7	100	100	100	100	100	100	65	50	0	0
	hMPV-A2	100	100	100	35	0	0	0	0	0	0
ABT 6-plex	H1N1pdm09	100	100	100	100	90	40	5	0	0	0
	H3N2	100	100	100	100	100	95	0	5	0	0
	B/Victoria	100	100	100	90	40	0	0	0	0	0
	RSVA	100	100	100	100	100	35	0	0	0	0
	RSVB	100	100	100	75	0	0	0	0	0	0
	PIV-1	100	100	100	100	80	30	10	0	0	0
	PIV-3	100	100	100	85	35	15	0	0	0	0
	HAdV-3	100	100	100	100	100	95	80	60	0	0
	HAdV-7	100	100	100	100	100	100	90	20	0	0
BioPerfectus 2-plex	H1N1pdm09	100	100	100	100	100	60	0	0	0	0
	H3N2	100	100	100	100	60	0	0	0	0	0
	B/Victoria	100	100	100	100	15	0	0	0	0	0

^*a*^ Omicron BA.5, severe acute respiratory syndrome coronavirus 2 Omicron BA.5 strain; H1N1pdm09, pandemic influenza A (H1N1) 2009 virus; H3N2, influenza A (H3N2) virus; B/Victoria, Victoria lineage of influenza B virus; HAdV-3, -7, human adenovirus type 3, 7; RSVA, respiratory syncytial virus subtype A; RSVB, respiratory syncytial virus subtype B; PIV-1, -2, -3, parainfluenza virus types 1, 2, 3; hMPV-A2, human metapneumovirus A2 strain; HRV-B72, human rhinovirus B72 strain.

### Assessment of competitive interference in co-infection of multiplex rRT-PCR kits

The results of competitive interference assays are presented in Table 3. The majority of the selected multiplex kits showed no competitive interference and adept recognition of co-infections, as evidenced by the expected positive rates of low-concentration analytes (approximately 3 × LOD) in co-infection samples. However, a positivity rate of 4 out of 20 in additional replicates using the BioPerfectus 2-Plex Kit indicated that a high concentration of H1N1pdm09 (approximately 1,000 × LOD) could affect the detection of B/Victoria at low concentrations (approximately 3 × LOD).

**TABLE 3:** Summary of competitive interference assays of multiplex kits^*d*^

Detection kit	Conditions		No. of detected/no. of replicates ^*a*^	Competitive interference (yes/no)	Average Ct value ± SD in co-infection samples^*b*^	Average Ct value ± SD in single-infection samples^*b*^
	Low-concentration analyte	High-concentration analyte				
Sansure 3-plex	H1N1pdm09	Omicron BA.5	3/3	No	29.88 ± 0.46	31.49 ± 0.14
	H1N1pdm09	B/Victoria	3/3	No	30.76 ± 0.05	31.49 ± 0.14
	H3N2	Omicron BA.5	3/3	No	30.17 ± 0.47	32.49 ± 0.07
	H3N2	B/Victoria	3/3	No	32.34 ± 0.17	32.49 ± 0.07
	B/Victoria	Omicron BA.5	3/3	No	32.09 ± 0.33	32.54 ± 0.37
	B/Victoria	H1N1pdm09	3/3	No	32.39 ± 0.45	32.54 ± 0.37
	B/Victoria	H3N2	3/3	No	32.80 ± 0.15	32.54 ± 0.37
	Omicron BA.5	B/Victoria	3/3	No	38.30 ± 1.19	37.47 ± 0.25
	Omicron BA.5	H1N1pdm09	3/3	No	37.82 ± 0.68	37.47 ± 0.25
	Omicron BA.5	H3N2	3/3	No	38.48 ± 1.01	37.47 ± 0.25
Sansure 6-plex	H1N1pdm09	RSVA	3/3	No	32.25 ± 0.12	34.05 ± 0.30
	H1N1pdm09	RSVB	3/3	No	33.37 ± 0.21	34.05 ± 0.30
	H1N1pdm09	B/Victoria	3/3	No	35.04 ± 0.24	34.05 ± 0.30
	H3N2	RSVA	3/3	No	34.35 ± 0.27	34.84 ± 0.17
	H3N2	RSVB	3/3	No	34.45 ± 0.16	34.84 ± 0.17
	H3N2	B/Victoria	3/3	No	35.78 ± 0.25	34.84 ± 0.17
	B/Victoria	H1N1pdm09	3/3	No	37.22 ± 1.17	37.47 ± 0.40
	B/Victoria	H3N2	3/3	No	37.24 ± 0.43	37.47 ± 0.40
	B/Victoria	RSVA	3/3	No	37.83 ± 0.87	37.47 ± 0.40
	B/Victoria	RSVB	17/20^c^	No	37.49 ± 1.20	37.83 ± 1.58
	RSVA	H1N1pdm09	3/3	No	31.03 ± 0.23	32.42 ± 0.26
	RSVA	H3N2	3/3	No	32.08 ± 0.13	32.42 ± 0.26
	RSVA	B/Victoria	3/3	No	32.27 ± 0.03	32.42 ± 0.26
	RSVB	H1N1pdm09	3/3	No	34.83 ± 0.20	34.69 ± 0.16
	RSVB	H3N2	3/3	No	34.72 ± 0.32	34.69 ± 0.16
	RSVB	B/Victoria	3/3	No	34.70 ± 0.24	34.69 ± 0.16
	HAdV-3	HRV-B72	3/3	No	37.60 ± 0.62	36.21 ± 0.31
	HAdV-7	HRV-B72	3/3	No	33.88 ± 0.27	33.83 ± 0.27
	HRV-B72	HAdV-3	3/3	No	35.92 ± 0.26	36.33 ± 0.39
	HRV-B72	HAdV-7	3/3	No	36.44 ± 0.47	36.33 ± 0.39
BioGerm 2-plex	H1N1pdm09	B/Victoria	3/3	No	31.75 ± 1.32	32.45 ± 0.97
	H3N2	B/Victoria	3/3	No	31.94 ± 0.35	32.38 ± 0.31
	B/Victoria	H1N1pdm09	3/3	No	32.70 ± 0.21	33.83 ± 0.30
	B/Victoria	H3N2	3/3	No	33.55 ± 0.32	33.83 ± 0.30
BioGerm 6-plex	PIV-1	hMPV-A2	3/3	No	32.12 ± 0.07	32.58 ± 0.14
	PIV-1	HAdV-3	3/3	No	31.59 ± 0.47	32.58 ± 0.14
	PIV-1	HAdV-7	3/3	No	33.19 ± 0.08	32.58 ± 0.14
	PIV-2	RSVA	3/3	No	32.53 ± 0.61	33.86 ± 0.28
	PIV-2	RSVB	3/3	No	32.54 ± 0.11	33.86 ± 0.28
	PIV-3	RSVA	3/3	No	29.60 ± 0.17	30.81 ± 2.04
	PIV-3	RSVB	3/3	No	30.08 ± 0.04	30.81 ± 2.04
	HAdV-3	hMPV-A2	3/3	No	34.82 ± 0.44	34.87 ± 0.30
	HAdV-3	PIV-1	20/20^*c*^	No	34.42 ± 0.64	34.81 ± 0.77
	HAdV-7	hMPV-A2	17/20^*c*^	No	35.02 ± 1.00	35.14 ± 0.86
	HAdV-7	PIV-1	3/3	No	35.85 ± 0.88	35.55 ± 0.21
	RSVA	PIV-2	3/3	No	36.00 ± 0.37	35.77 ± 0.18
	RSVA	PIV-3	3/3	No	35.46 ± 0.06	35.77 ± 0.18
	RSVB	PIV-2	3/3	No	34.68 ± 0.06	33.24 ± 0.14
	RSVB	PIV-3	3/3	No	33.96 ± 0.32	33.24 ± 0.14
	hMPV-A2	PIV-1	3/3	No	34.89 ± 0.71	35.17 ± 0.31
	hMPV-A2	HAdV-3	3/3	No	33.26 ± 0.31	35.17 ± 0.31
	hMPV-A2	HAdV-7	3/3	No	35.11 ± 0.30	35.17 ± 0.31
ABT 6-pelx	H1N1pdm09	RSVA	3/3	No	31.75 ± 0.25	33.48 ± 0.13
	H1N1pdm09	RSVB	3/3	No	33.27 ± 0.19	33.48 ± 0.13
	H1N1pdm09	HAdV-3	3/3	No	32.25 ± 0.15	33.48 ± 0.13
	H1N1pdm09	HAdV-7	3/3	No	31.96 ± 0.19	33.48 ± 0.13
	H3N2	RSVA	3/3	No	31.72 ± 0.11	33.09 ± 0.13
	H3N2	RSVB	3/3	No	32.79 ± 0.27	33.09 ± 0.13
	H3N2	HAdV-3	3/3	No	32.13 ± 0.16	33.09 ± 0.13
	H3N2	HAdV-7	3/3	No	29.83 ± 0.05	33.09 ± 0.13
	B/Victoria	PIV-1	3/3	No	31.89 ± 0.13	33.33 ± 0.14
	B/Victoria	PIV-3	3/3	No	33.56 ± 0.53	33.33 ± 0.14
	RSVA	H1N1pdm09	3/3	No	32.30 ± 0.10	32.74 ± 0.07
	RSVA	H3N2	3/3	No	32.29 ± 0.12	32.74 ± 0.07
	RSVA	HAdV-3	3/3	No	31.25 ± 1.58	32.74 ± 0.07
	RSVA	ADV7	3/3	No	32.76 ± 0.14	32.74 ± 0.07
	RSVB	H1N1pdm09	3/3	No	32.97 ± 0.20	33.11 ± 0.29
	RSVB	H3N2	3/3	No	32.83 ± 0.34	33.11 ± 0.29
	RSVB	HAdV-3	3/3	No	32.39 ± 0.40	33.11 ± 0.29
	RSVB	HAdV-7	3/3	No	32.52 ± 0.09	33.11 ± 0.29
	HAdV-3	H1N1pdm09	3/3	No	30.95 ± 0.07	31.93 ± 0.15
	HAdV-3	H3N2	3/3	No	31.10 ± 0.43	31.93 ± 0.15
	HAdV-3	RSVA	3/3	No	31.24 ± 0.08	31.93 ± 0.15
	HAdV-3	RSVB	3/3	No	31.66 ± 0.30	31.93 ± 0.15
	HAdV-7	H1N1pdm09	3/3	No	31.97 ± 0.14	32.59 ± 0.16
	HAdV-7	H3N2	3/3	No	32.04 ± 0.10	32.59 ± 0.16
	HAdV-7	RSVA	3/3	No	32.54 ± 0.10	32.59 ± 0.16
	HAdV-7	RSVB	3/3	No	32.64 ± 0.29	32.59 ± 0.16
	PIV-1	B/Victoria	3/3	No	33.17 ± 0.04	32.91 ± 0.05
	PIV-1	PIV-3	3/3	No	32.88 ± 0.06	32.91 ± 0.05
	PIV-3	B/Victoria	3/3	No	30.11 ± 0.18	32.14 ± 0.35
	PIV-3	PIV-1	3/3	No	28.35 ± 0.07	32.14 ± 0.35
BioPerfectus 2-plex	H1N1pdm09	B/Victoria	3/3	No	32.62 ± 0.54	32.19 ± 0.33
	H3N2	B/Victoria	3/3	No	33.79 ± 1.57	32.33 ± 0.47
	B/Victoria	H1N1pdm09	4/20^*c*^	Yes	35.40 ± 0.64	33.38 ± 0.25
	B/Victoria	H3N2	3/3	No	33.17 ± 0.30	33.27 ± 0.14

^*a*^ Positive rate of low-concentration analyte in co-infection samples.

^*b*^ Average Ct value ± SD of low-concentration analyte.

^*c*^ A supplementary validation procedure of 20 additional replicates was conducted.

^*d*^ Omicron BA.5, severe acute respiratory syndrome coronavirus 2 Omicron BA.5 strain; H1N1pdm09, pandemic influenza A (H1N1) 2009 virus; H3N2, influenza A (H3N2) virus; B/Victoria, Victoria lineage of influenza B virus; HAdV-3, -7, human adenovirus type 3, 7; RSVA, respiratory syncytial virus subtype A; RSVB, respiratory syncytial virus subtype B; PIV-1, -2, -3, parainfluenza virus types 1, 2, 3; hMPV-A2, human metapneumovirus A2 strain; HRV-B72, human rhinovirus B72 strain; Ct, cycle threshold; SD, standard deviation.

## DISCUSSION

Viral RTIs are extremely frequent in adults and children, representing an increased burden on the healthcare systems (13). Diagnostic underestimation of viral RTIs poses the risk of irrational treatment and viral transmission (36). Advanced technology and enhanced diagnostic capabilities have catalyzed a paradigm shift in the molecular diagnosis of viral RTIs, enabling the widespread development and application of multiplex rRT-PCR assays (10, 19, 20).

Analytical sensitivity is a key performance parameter, and it varied for different kits evaluated in this study. The critical factors influencing and determining analytical sensitivity are typically linked to the initial design of kits for the entire testing process, encompassing nucleic acid preparation, reaction components, and reaction conditions (14, 23). During the process of viral nucleic acid preparation, sample input and elution volumes and template and reaction volumes were different (Table S3), both of which could affect nucleic acid concentration and detection performance of the kit (37). The presence of multiple primer pairs and higher concentrations of reaction components, such as dNTPs, buffer, and enzymes, in multiplex assays may increase the likelihood of primer dimer formation or mis-priming, with the subsequent production of spurious nonspecific amplification products compared with singleplex assays, compromising the binding of desired targets (17, 18, 23, 38–41). Unlike multiple singleplex assays for different targets, identical temperatures and recycling times are set for different targets in multiplex assays, and therefore, amplification efficiency differed between singleplex and multiplex assays (42).

However, variable analytical sensitivity across viruses using the same multiplex kit indicates that other factors may affect analytical sensitivity. PCR selection is an unbalanced amplification mechanism in multiplex assays (43). PCR selection encompasses the inherent properties of the target, such as preferential denaturation resulting from overall low GC content, increased efficiency of GC-rich primer-binding regions, and differential accessibility of targets within the genome due to secondary structures, gene copy numbers, and other related factors (17). In addition, mutation in subtypes of the virus is an important impact factor; C154T, G225A, T228C, and G238A mutations present in H1N1pmd09, and C153T, C163T, and G189T mutations present in H3N2 have reduced the sensitivity of many commercial kits (44–46). Thus, unbalanced amplification caused by PCR selection of viruses and potential mutations in virus subtypes inherently favors the amplification of certain target templates within multiplex kits.

Unbalanced amplification may subsequently cause competitive interference in scenarios of co-infections. We primarily focused on evaluating the analytical performance of competitive interference under specific extreme conditions because competitive interference becomes more pronounced with greater differences in the concentrations of the two analytes during co-infection (26, 27, 35). One case of competitive interference was observed when an interfering analyte was present at high concentrations (approximately 1,000 × LOD) in the co-infected sample. Considering that co-infection of two analytes with significantly different concentrations is rare in clinical practice, the risk of interference using multiplex kits should be very low (47).

Based on these results, improvements can be made in the design of multiplex rRT-PCR kits from multiple perspectives. First, the entire testing process cannot be ignored. Appropriately increasing the sample input volume and template volume in the reaction enhances the detection of low-concentration analytes in the sample (14). Second, the meticulous design of primers and probes is paramount (48, 49). Researchers must not only prioritize the homology between primers and their target but also steer clear of substantial homology either internally or among themselves to reduce primer dimers and avoid PCR selection (42, 49). Special attention should be paid to high-frequency mutations in viruses and selection from conserved regions of the genome (44). Despite the availability of software tools for predicting the performance of multiplex rRT-PCR assays, comprehensive validation and verification may be necessary (38, 48, 50–54). Finally, modifying the reagent components and adjusting the reaction conditions can yield advantageous outcomes (42, 55).

When implementing rRT-PCR kits, careful consideration should be given to balance detection performance and clinical significance (8, 13). According to the results, through variable LODs across viruses and kits, all the tested kits showed a 100% positivity rate when the viral concentration exceeded 30,000 copies/mL, proving their effectiveness in detecting viral RTIs (47, 56–60). We support preferential consideration of multiplex rRT-PCR kits for RTIs where there is a notable prevalence of influenza virus with other respiratory viruses in circulation, such as SARS-CoV-2, RSV, and HAdV, whereas singleplex kits may be more suitable for the detection of HRV, hMPV, and PIV. In addition, a thorough analysis of the test results of rRT-PCR kits with clinical features and detection performance is imperative. In the case of conflicting results, verification can be conducted using alternative detection kits that have been evaluated to offer superior sensitivity and specificity.

This research highlights the differences in analytical performance between currently available multiplex and singleplex assays, as well as the variations in target detection performance of multiplex kits, providing valuable insights for optimization and application. However, this study has a few limitations. FDA-approved tests were not included because of limited usage in China, which may have affected the generalizability of our study. The viral samples employed in our study did not represent all characteristics of clinical patient samples. There might be potential impact of using PBS as a uniform preservation solution on the performance of specific detection kits. Thus, the conclusions regarding the analytical sensitivity and competitive interference were specific to the viral samples used in this study.

In conclusion, we evaluated the analytical sensitivity and competitive interference of the top six multiplex rRT-PCR kits for detecting respiratory viruses prevalent in China. Despite some shortcomings, unavoidable challenges, and optimization complexities, multiplex rRT-PCR assays have potential as valuable diagnostic tools for viral RTIs.

## SUPPLEMENTAL MATERIAL

Tables S1 to S3 spectrum.01641-24-s0001.docx Additional experimental details.

ASM does not own the copyrights to Supplemental Material that may be linked to, or accessed through, an article. The authors have granted ASM a non-exclusive, world-wide license to publish the Supplemental Material files. Please contact the corresponding author directly for reuse.

## References

[1] Williams BG, Gouws E, Boschi-Pinto C, Bryce J, Dye C. 2002. Estimates of world-wide distribution of child deaths from acute respiratory infections. Lancet Infect Dis 2:25–32. doi:10.1016/s1473-3099(01)00170-0 11892493

[2] Moriyama M, Hugentobler WJ, Iwasaki A. 2020. Seasonality of respiratory viral infections. Annu Rev Virol 7:83–101. doi:10.1146/annurev-virology-012420-022445 32196426

[3] Li Z-J, Zhang H-Y, Ren L-L, Lu Q-B, Ren X, Zhang C-H, Wang Y-F, Lin S-H, Zhang X-A, Li J, et al.. 2021. Etiological and epidemiological features of acute respiratory infections in China. Nat Commun 12:5026. doi:10.1038/s41467-021-25120-6 34408158 PMC8373954

[4] Abdelrahman Z, Li M, Wang X. 2020. Comparative review of SARS-CoV-2, SARS-CoV, MERS-CoV, and influenza A respiratory viruses. Front Immunol 11:552909. doi:10.3389/fimmu.2020.552909 33013925 PMC7516028

[5] Clark TW, Lindsley K, Wigmosta TB, Bhagat A, Hemmert RB, Uyei J, Timbrook TT. 2023. Rapid multiplex PCR for respiratory viruses reduces time to result and improves clinical care: results of a systematic review and meta-analysis. J Infect 86:462–475. doi:10.1016/j.jinf.2023.03.005 36906153

[6] Qian Y, Ai J, Wu J, Yu S, Cui P, Gao Y, Jin J, Weng X, Zhang W. 2020. Rapid detection of respiratory organisms with FilmArray respiratory panel and its impact on clinical decisions in Shanghai, China, 2016-2018. Influenza Other Respir Viruses 14:142–149. doi:10.1111/irv.12701 31786832 PMC7040966

[7] Zhang N, Wang L, Deng X, Liang R, Su M, He C, Hu L, Su Y, Ren J, Yu F, Du L, Jiang S. 2020. Recent advances in the detection of respiratory virus infection in humans. J Med Virol 92:408–417. doi:10.1002/jmv.25674 31944312 PMC7166954

[8] Vallières E, Renaud C. 2013. Clinical and economical impact of multiplex respiratory virus assays. Diagn Microbiol Infect Dis 76:255–261. doi:10.1016/j.diagmicrobio.2013.03.008 23601453 PMC7132665

[9] Mackay IM, Arden KE, Nitsche A. 2002. Real-time PCR in virology. Nucleic Acids Res 30:1292–1305. doi:10.1093/nar/30.6.1292 11884626 PMC101343

[10] Pabbaraju K, Wong S, Lee B, Tellier R, Fonseca K, Louie M, Drews SJ. 2011. Comparison of A singleplex real-time RT-PCR assay and multiplex respiratory viral panel assay for detection of influenza “A” in respiratory specimens. Influenza Other Respir Viruses 5:99–103. doi:10.1111/j.1750-2659.2010.00170.x 21244644 PMC4942004

[11] Bogdan I, Gadela T, Bratosin F, Dumitru C, Popescu A, Horhat FG, Negrean RA, Horhat RM, Mot IC, Bota AV, Stoica CN, Feciche B, Csep AN, Fericean RM, Chicin GN, Marincu I. 2023. The assessment of multiplex PCR in identifying bacterial infections in patients hospitalized with SARS-CoV-2 infection: a systematic review. Antibiotics (Basel) 12:465. doi:10.3390/antibiotics12030465 36978332 PMC10044563

[12] Huang HS, Tsai CL, Chang J, Hsu TC, Lin S, Lee CC. 2018. Multiplex PCR system for the rapid diagnosis of respiratory virus infection: systematic review and meta-analysis. Clin Microbiol Infect 24:1055–1063. doi:10.1016/j.cmi.2017.11.018 29208560 PMC7128951

[13] Niederman MS, Torres A. 2022. Respiratory infections. Eur Respir Rev 31:220150. doi:10.1183/16000617.0150-2022 36261160 PMC9724828

[14] Yang J, Li D, Wang J, Zhang R, Li J. 2022. Design, optimization, and application of multiplex rRT-PCR in the detection of respiratory viruses. Crit Rev Clin Lab Sci 59:555–572. doi:10.1080/10408363.2022.2072467 35559711

[15] Muthuri SG, Venkatesan S, Myles PR, Leonardi-Bee J, Al Khuwaitir TSA, Al Mamun A, Anovadiya AP, Azziz-Baumgartner E, Báez C, Bassetti M, et al.. 2014. Effectiveness of neuraminidase inhibitors in reducing mortality in patients admitted to hospital with influenza A H1N1pdm09 virus infection: a meta-analysis of individual participant data. Lancet Respir Med 2:395–404. doi:10.1016/S2213-2600(14)70041-4 24815805 PMC6637757

[16] Greenberg A, Barish P, Hoffman A. 2020. Overuse of respiratory viral panels: a teachable moment. JAMA Intern Med 180:1373–1374. doi:10.1001/jamainternmed.2020.3535 32865562

[17] Elnifro EM, Ashshi AM, Cooper RJ, Klapper PE. 2000. Multiplex PCR: optimization and application in diagnostic virology. Clin Microbiol Rev 13:559–570. doi:10.1128/CMR.13.4.559 11023957 PMC88949

[18] Shum J, Paul N. 2009. Chemically modified primers for improved multiplex polymerase chain reaction. Anal Biochem 388:266–272. doi:10.1016/j.ab.2009.02.033 19258004 PMC2716723

[19] Sakthivel SK, Whitaker B, Lu X, Oliveira DBL, Stockman LJ, Kamili S, Oberste MS, Erdman DD. 2012. Comparison of fast-track diagnostics respiratory pathogens multiplex real-time RT-PCR assay with in-house singleplex assays for comprehensive detection of human respiratory viruses. J Virol Methods 185:259–266. doi:10.1016/j.jviromet.2012.07.010 22796035 PMC7119496

[20] Onwuchekwa C, Moreo LM, Menon S, Machado B, Curcio D, Kalina W, Atwell JE, Gessner BD, Siapka M, Agarwal N, Rubbrecht M, Nair H, Rozenbaum M, Aponte-Torres Z, Vroling H, Begier E. 2023. Underascertainment of respiratory syncytial virus infection in adults due to diagnostic testing limitations: a systematic literature review and meta-analysis. J Infect Dis 228:173–184. doi:10.1093/infdis/jiad012 36661222 PMC10345483

[21] Walls T, McSweeney A, Anderson T, Jennings LC. 2017. Multiplex-PCR for the detection of viruses in the CSF of infants and young children. J Med Virol 89:559–561. doi:10.1002/jmv.24461 26702584

[22] Wolters F, Grünberg M, Huber M, Kessler HH, Prüller F, Saleh L, Fébreau C, Rahamat-Langendoen J, Thibault V, Melchers WJG. 2021. European multicenter evaluation of Xpert Xpress SARS-CoV-2/Flu/RSV test. J Med Virol 93:5798–5804. doi:10.1002/jmv.27111 34050951 PMC8242864

[23] Ni M, Xu H, Luo J, Liu W, Zhou D. 2021. Simultaneous detection and differentiation of SARS-CoV-2, influenza A virus and influenza B virus by one-step quadruplex real-time RT-PCR in patients with clinical manifestations. Int J Infect Dis 103:517–524. doi:10.1016/j.ijid.2020.12.027 33326873 PMC7836965

[24] Li Y, Wang X, Blau DM, Caballero MT, Feikin DR, Gill CJ, Madhi SA, Omer SB, Simões EAF, Campbell H, et al.. 2022. Global, regional, and national disease burden estimates of acute lower respiratory infections due to respiratory syncytial virus in children younger than 5 years in 2019: a systematic analysis. Lancet 399:2047–2064. doi:10.1016/S0140-6736(22)00478-0 35598608 PMC7613574

[25] Olofsson S, Brittain-Long R, Andersson LM, Westin J, Lindh M. 2011. PCR for detection of respiratory viruses: seasonal variations of virus infections. Expert Rev Anti Infect Ther 9:615–626. doi:10.1586/eri.11.75 21819328 PMC7103711

[26] U.S. Food and Drug Administration. 2023. 510(k) premarket notification. Available from: https://www.accessdata.fda.gov/scripts/cdrh/cfdocs/cfPMN/pmn.cfm?ID=K131813. Retrieved 5 Jun 2024.

[27] U.S. Food and Drug Administration. 2013. 510(k) premarket notification. Available from: https://www.accessdata.fda.gov/scripts/cdrh/cfdocs/cfPMN/pmn.cfm?ID=K122189. Retrieved 5 Jun 2021.

[28] Butt SA, Maceira VP, McCallen ME, Stellrecht KA. 2014. Comparison of three commercial RT-PCR systems for the detection of respiratory viruses. J Clin Virol 61:406–410. doi:10.1016/j.jcv.2014.08.010 25183359 PMC7172935

[29] Zou X, Chang K, Wang Y, Li M, Zhang W, Wang C, Lu B, Xiong Z, Han J, Zhang Y, Zhao J, Cao B. 2019. Comparison of the Cepheid Xpert Xpress Flu/RSV assay and commercial real-time PCR for the detection of influenza A and influenza B in A prospective cohort from China. Int J Infect Dis 80:92–97. doi:10.1016/j.ijid.2018.12.014 30634045

[30] Gosert R, Naegele K, Hirsch HH. 2019. Comparing the Cobas Liat Influenza A/B and respiratory syncytial virus assay with multiplex nucleic acid testing. J Med Virol 91:582–587. doi:10.1002/jmv.25344 30345524 PMC7166997

[31] Stellrecht KA, Cimino JL, Wilson LI, Maceira VP, Butt SA. 2019. Panther Fusion Respiratory Virus Assays for the detection of influenza and other respiratory viruses. J Clin Virol 121:104204. doi:10.1016/j.jcv.2019.104204 31743836 PMC7172166

[32] U.S. Food and Drug Administration. 2009. Respiratory viral panel multiplex nucleic acid assay - class II special controls guidance for industry and FDA staff. Available from: https://www.fda.gov/medical-devices/guidance-documents-medical-devices-and-radiation-emitting-products/respiratory-viral-panel-multiplex-nucleic-acid-assay-class-ii-special-controls-guidance-industry-and. Retrieved 5 Jun 2024.

[33] ISO. 2022. In vitro diagnostic medical devices—multiplex molecular testing for nucleic acids—part 2: validation and verification. Available from: https://www.iso.org/standard/78024.html. Retrieved 5 Jun 2024.

[34] Chen Y, Feng L, Han Y, Zhao Z, Diao Z, Huang T, Ma Y, Feng W, Li J, Li Z, Liu C, Chang L, Li J, Zhang R. 2023. Performance evaluation of SARS-CoV-2 antigen detection in the post-pandemic era: multi-laboratory assessment. Clin Chem Lab Med 61:2237–2247. doi:10.1515/cclm-2023-0597 37377068

[35] U.S. Food and Drug Administration. 2017. 510(k) premarket notification. Available from: https://www.accessdata.fda.gov/scripts/cdrh/cfdocs/cfPMN/pmn.cfm?ID=K162331. Retrieved 5 Jun 2024.

[36] Cia C-T, Lin I-T, Lee J-C, Tsai H-P, Wang J-R, Ko W-C. 2021. Respiratory viral infections in pragmatically selected adults in intensive care units. Sci Rep 11:20058. doi:10.1038/s41598-021-99608-y 34625621 PMC8501073

[37] Yang J, Han Y, Zhang R, Zhang R, Li J. 2021. Comparison of analytical sensitivity of SARS-CoV-2 molecular detection kits. Int J Infect Dis 111:233–241. doi:10.1016/j.ijid.2021.08.043 34428543 PMC8379823

[38] Xie NG, Wang MX, Song P, Mao S, Wang Y, Yang Y, Luo J, Ren S, Zhang DY. 2022. Designing highly multiplex PCR primer sets with simulated annealing design using dimer likelihood estimation (SADDLE). Nat Commun 13:1881. doi:10.1038/s41467-022-29500-4 35410464 PMC9001684

[39] Koukhareva I, Lebedev A. 2009. 3’-Protected 2’-deoxynucleoside 5’-triphosphates as a tool for heat-triggered activation of polymerase chain reaction. Anal Chem 81:4955–4962. doi:10.1021/ac8026977 19438248 PMC2712722

[40] Raeymaekers L. 1995. A commentary on the practical applications of competitive PCR. Genome Res 5:91–94. doi:10.1101/gr.5.1.91 8717060

[41] Chamberlain JS, Gibbs RA, Ranier JE, Nguyen PN, Caskey CT. 1988. Deletion screening of the Duchenne muscular dystrophy locus via multiplex DNA amplification. Nucleic Acids Res 16:11141–11156. doi:10.1093/nar/16.23.11141 3205741 PMC339001

[42] Henegariu O, Heerema NA, Dlouhy SR, Vance GH, Vogt PH. 1997. Multiplex PCR: critical parameters and step-by-step protocol. BioTechniques 23:504–511. doi:10.2144/97233rr01 9298224

[43] Yun J, Park JH, Kim N, Roh EY, Shin S, Yoon JH, Kim TS, Park H. 2021. Evaluation of three multiplex real-time reverse transcription PCR assays for simultaneous detection of SARS-CoV-2, Influenza A/B, and respiratory syncytial virus in nasopharyngeal swabs. J Korean Med Sci 36:e328. doi:10.3346/jkms.2021.36.e328 34904407 PMC8668494

[44] Chen Y, Han Y, Yang J, Ma Y, Li J, Zhang R. 2022. Impact of SARS-CoV-2 variants on the analytical sensitivity of rRT-PCR assays. J Clin Microbiol 60:e0237421. doi:10.1128/jcm.02374-21 35341301 PMC9020341

[45] Stellrecht KA. 2018. The drift in molecular testing for influenza: mutations affecting assay performance. J Clin Microbiol 56:e01531-17. doi:10.1128/JCM.01531-17 29305549 PMC5824055

[46] Stellrecht KA, Nattanmai SM, Butt J, Maceira VP, Espino AA, Castro AJ, Landes A, Dresser N, Butt SA. 2017. Effect of genomic drift of influenza PCR tests. J Clin Virol 93:25–29. doi:10.1016/j.jcv.2017.05.016 28600949 PMC7173001

[47] Granados A, Peci A, McGeer A, Gubbay JB. 2017. Influenza and rhinovirus viral load and disease severity in upper respiratory tract infections. J Clin Virol 86:14–19. doi:10.1016/j.jcv.2016.11.008 27893998

[48] Rodríguez A, Rodríguez M, Córdoba JJ, Andrade MJ. 2015. Design of primers and probes for quantitative real-time PCR methods. Methods Mol Biol 1275:31–56. doi:10.1007/978-1-4939-2365-6_3 25697650

[49] Suzuki MT, Giovannoni SJ. 1996. Bias caused by template annealing in the amplification of mixtures of 16S rRNA genes by PCR. Appl Environ Microbiol 62:625–630. doi:10.1128/aem.62.2.625-630.1996 8593063 PMC167828

[50] Rachlin J, Ding C, Cantor C, Kasif S. 2005. MuPlex: multi-objective multiplex PCR assay design. Nucleic Acids Res 33:W544–7. doi:10.1093/nar/gki377 15980531 PMC1160138

[51] Shen Z, Qu W, Wang W, Lu Y, Wu Y, Li Z, Hang X, Wang X, Zhao D, Zhang C. 2010. MPprimer: a program for reliable multiplex PCR primer design. BMC Bioinformatics 11:143. doi:10.1186/1471-2105-11-143 20298595 PMC2858037

[52] Park M, Won J, Choi BY, Lee CJ. 2020. Optimization of primer sets and detection protocols for SARS-CoV-2 of coronavirus disease 2019 (COVID-19) using PCR and real-time PCR. Exp Mol Med 52:963–977. doi:10.1038/s12276-020-0452-7 32546849 PMC7295692

[53] Li Y, Guo SJ, Shao N, Tu S, Xu M, Ren ZR, Ling X, Wang GQ, Lin ZX, Tao SC. 2011. A universal multiplex PCR strategy for 100-plex amplification using A hydrophobically patterned microarray. Lab Chip 11:3609–3618. doi:10.1039/c1lc20526a 21909519

[54] Bustin SA, Benes V, Garson JA, Hellemans J, Huggett J, Kubista M, Mueller R, Nolan T, Pfaffl MW, Shipley GL, Vandesompele J, Wittwer CT. 2009. The MIQE guidelines: minimum information for publication of quantitative real-time PCR experiments. Clin Chem 55:611–622. doi:10.1373/clinchem.2008.112797 19246619

[55] Markoulatos P, Siafakas N, Moncany M. 2002. Multiplex polymerase chain reaction: a practical approach. J Clin Lab Anal 16:47–51. doi:10.1002/jcla.2058 11835531 PMC6808141

[56] Franz A, Adams O, Willems R, Bonzel L, Neuhausen N, Schweizer-Krantz S, Ruggeberg JU, Willers R, Henrich B, Schroten H, Tenenbaum T. 2010. Correlation of viral load of respiratory pathogens and co-infections with disease severity in children hospitalized for lower respiratory tract infection. J Clin Virol 48:239–245. doi:10.1016/j.jcv.2010.05.007 20646956 PMC7185496

[57] To KK-W, Tsang OT-Y, Leung W-S, Tam AR, Wu T-C, Lung DC, Yip CC-Y, Cai J-P, Chan JM-C, Chik TS-H, Lau DP-L, Choi CY-C, Chen L-L, Chan W-M, Chan K-H, Ip JD, Ng AC-K, Poon RW-S, Luo C-T, Cheng VC-C, Chan JF-W, Hung IF-N, Chen Z, Chen H, Yuen K-Y. 2020. Temporal profiles of viral load in posterior oropharyngeal saliva samples and serum antibody responses during infection by SARS-CoV-2: an observational cohort study. Lancet Infect Dis 20:565–574. doi:10.1016/S1473-3099(20)30196-1 32213337 PMC7158907

[58] Lee N, Chan PKS, Hui DSC, Rainer TH, Wong E, Choi K-W, Lui GCY, Wong BCK, Wong RYK, Lam W-Y, Chu IMT, Lai RWM, Cockram CS, Sung JJY. 2009. Viral loads and duration of viral shedding in adult patients hospitalized with influenza. J Infect Dis 200:492–500. doi:10.1086/600383 19591575 PMC7110250

[59] Martin ET, Kuypers J, Wald A, Englund JA. 2012. Multiple versus single virus respiratory infections: viral load and clinical disease severity in hospitalized children. Influenza Other Respir Viruses 6:71–77. doi:10.1111/j.1750-2659.2011.00265.x 21668660 PMC3175338

[60] Fairchok MP, Martin ET, Chambers S, Kuypers J, Behrens M, Braun LE, Englund JA. 2010. Epidemiology of viral respiratory tract infections in a prospective cohort of infants and toddlers attending daycare. J Clin Virol 49:16–20. doi:10.1016/j.jcv.2010.06.013 20650679 PMC7108368

