# Supplementary material for: Key performance evaluation of commercialized multiplex rRT-PCR kits for respiratory viruses: implications for application and optimization
Source: Microbiol Spectr. 2024 Oct 29;12(12):e01641-24. doi: 10.1128/spectrum.01641-24 (PMC11619282; doi:10.1128/spectrum.01641-24)
Supplement: Tables S1 to S3 — Additional experimental details. [file spectrum.01641-24-s0001.docx]

**Table S1** Nucleotide sequences of primers and probes used for digital PCR assays and RT-qPCR assays.

| **Virus samples** | **Sequence (5′-3′)** | | **Gene target** | **Reference** |
| --- | --- | --- | --- | --- |
| hMPV-A2 | Forward primer | CATACAAGCATGCTATATTAAAAGAGTCTC | Nucleoprotein gene | (1) |
|  | Reverse primer | CCTATTTCTGCAGCATATTTGTAATCAG |  |  |
|  | TaqMan probe | TGCAATGATGAGGGTGTCACTGCTGTTG |  |  |
| B/Victoria | Forward primer | TCCTCAACTCACTCTTCGAGCG | Nonstructural protein gene | (2) |
|  | Reverse primer | CGGTGCTCTTGACCAAATTGG |  |  |
|  | TaqMan probe | CCAATTCGAGCAGCTGAAACTGCGGTG |  |  |
| H3N2 | Forward primer | GACCAATCCTGTCACCTCTGAC | Matrix gene | (3) |
|  | Reverse primer | AGGGCATTTTGGACAAAGCGTCTA |  |  |
|  | TaqMan probe | TGCAGTCCTCGCTCACTGGGCACG |  |  |
| H1N1pdm09 | Forward primer | GACCAATCTTGTCACCTCTGAC | Matrix gene | (3) |
|  | Reverse primer | AGGGCATTTTGGACAAAGCGTCTA |  |  |
|  | TaqMan probe | TGCAGTCCTCGCTCACTGGGCACG |  |  |
| Omicron BA.5 | Forward primer | AACGAACTTCTCCTGCTAGAAT | Nucleocapsid gene | (4) |
|  | Reverse primer | CAGACATTTTGCTCTCAAGCTG |  |  |
|  | TaqMan probe | TTGCTGCTGCTTGACAGATT |  |  |
| RSVA | Forward primer | GCTCTTAGCAAAGTCAAGTTGAATGA | Nucleoprotein gene | (5) |
|  | Reverse primer | TGCTCCGTTGGATGGTGTATT |  |  |
|  | TaqMan probe | ACACTCAACAAAGATCAACTTCTGTCATCTAGC |  |  |
| RSVB | Forward primer | GATGGCTCTTAGCAAAGTCAAGTTGA | Nucleoprotein gene | (5) |
|  | Reverse primer | TGTCAATATTATCTCCTGTACTACGTTGAA |  |  |
|  | TaqMan probe | TGATACATTAAATAAGGATCAGCTGCTGTCATCCA |  |  |
| HAdV-3 | Forward primer | GCCACTGTGGGGTTTCTAAATTT | Hexon capsid protein gene | (6) |
|  | Reverse primer | GCCCCAATGGGCATACATGCACATC |  |  |
|  | TaqMan probe | TGCACCAGACCCGGACTCAGGTACTCCGA |  |  |
| HAdV-7 | Forward primer | GCCACTGTGGGGTTTCTAAACTT | Hexon capsid protein gene | (6) |
|  | Reverse primer | GCCCCAATGGGCATACATGCACATC |  |  |
|  | TaqMan probe | TGCACCAGACCCGGACTCAGGTACTCCGA |  |  |
| PIV-1 | Forward primer | TGATTTAAAACCGGTAATTTCTCAT | Hemagglutinin-neuraminidase gene | (7) |
|  | Reverse primer | CCTTGTTCCTGCAGCTATTACAGA |  |  |
|  | TaqMan probe | ACGACAACAGGAAATC |  |  |
| PIV-2 | Forward primer | AGGACTATGAAAACCATTTACCTAAGTGA | Hemagglutinin-neuraminidase gene | (7) |
|  | Reverse primer | AAGCAAGTCTCAGTTCAGCTAGATCA |  |  |
|  | TaqMan probe | ATCAATCGCAAAAGCTGTTCAGTCACTGCTATAC |  |  |
| PIV-3 | Forward primer | TGATGAAAGATCAGATTATGCATCATC | Hemagglutinin-neuraminidase gene | (7) |
|  | Reverse primer | CCAGGACACCCAGTTGTG |  |  |
|  | TaqMan probe | TGGACCAGGGATATACTACAAAGGCAAAATAATATTTCTC |  |  |
| HRV-B72 | Forward primer | CCAGCCTGCGTGGC | 5'-untranslated region | (8) |
|  | Reverse primer | GAAACACGGACACCCAAAGTA |  |  |
|  | TaqMan probe | TCCGCCGGCCCCTGAATGCGGC |  |  |

Abbreviations: Omicron BA.5, severe acute respiratory syndrome coronavirus 2 Omicron BA.5 strain; H1N1pdm09, pandemic influenza A (H1N1) 2009 virus; H3N2, influenza A (H3N2) virus; B/Victoria, Victoria-lineage of influenza B virus; HAdV-3, -7, human adenovirus type 3, 7; RSVA, respiratory syncytial virus subtypes A; RSVB, respiratory syncytial virus subtypes B; PIV-1, -2, -3, parainfluenza virus type 1, 2, 3; hMPV-A2, human metapneumovirus A2 strain; HRV-B72, human rhinovirus B72 strain.

**Table S2** The result of matrix equivalency study

| **Detection kit** |  | **Sansure 3-plex**  **Omicron BA.5**  **near 3×LOD** | |  | **BioGerm 6-plex**  **HAdV-3**  **near 3×LOD** | |  | **ABT 6-plex**  **HAdV-3**  **near 3×LOD** | |  | **Daan singleplex**  **Omicron BA.5**  **near 3×LOD** | |
| --- | --- | --- | --- | --- | --- | --- | --- | --- | --- | --- | --- | --- |
| **Matrix types** |  | **simulated clinical matrix** | **PBS** |  | **simulated clinical matrix** | **PBS** |  | **simulated clinical matrix** | **PBS** |  | **simulated clinical matrix** | **PBS** |
| Average Ct values |  | 36.85 | 37.02 |  | 35.35 | 35.36 |  | 31.89 | 31.87 |  | 36.12 | 35.94 |
| SD |  | 0.72 | 0.64 |  | 1.21 | 0.96 |  | 0.22 | 0.21 |  | 0.83 | 0.57 |
| Positivity rate |  | 20/20 | 20/20 |  | 20/20 | 20/20 |  | 20/20 | 20/20 |  | 20/20 | 20/20 |
| Delta Ct |  | 0.17 | |  | 0.01 | |  | 0.02 | |  | 0.18 | |
| Delta Positivity |  | 0 | |  | 0 | |  | 0 | |  | 0 | |
| P value |  | 0.43 | |  | 0.99 | |  | 0.78 | |  | 0.48 | |

Abbreviations: Omicron BA.5, severe acute respiratory syndrome coronavirus 2 Omicron BA.5 strain; HAdV-3, human adenovirus type 3; LOD, limit od detections; PBS, phosphate buffered saline; Ct, cycle threshold; SD, Standard deviation.

**Table S3** Characteristics of viral nucleic acid preparation for selected rRT-PCR kits.

| **Virus samples** | **Detection kits** | **Input Vol. (μl)** | **Elution Vol. (μl)** | **Template Vol. (μl)** | **Total reaction Vol. (μl)** |
| --- | --- | --- | --- | --- | --- |
| Omicron BA.5 | Sansure 3-plex | 300 | 80 | 20 | 50 |
|  | Sansure singleplex | 300 | 80 | 20 | 50 |
|  | BioGerm singleplex | 300 | / | 20 | 40 |
|  | Daan singleplex | 200 | 100 | 20 | 40 |
| H1N1pdm09, H3N2 | Sansure 6-plex | 300 | 80 | 5 | 50 |
|  | Sansure 3-plex | 300 | 80 | 20 | 50 |
|  | Sansure singleplex | 300 | 80 | 9 | 50 |
|  | ABT 6-plex | 200 | 60 | 5 | 25 |
|  | BioGerm 2-plex | 300 | / | 5 | 25 |
|  | BioPerfectus 2-plex | 140 | 60 | 5 | 25 |
|  | Daan singleplex | 200 | 100 | 5 | 25 |
| B/Victoria | Sansure 6-plex | 300 | 80 | 5 | 50 |
|  | Sansure 3-plex | 300 | 80 | 20 | 50 |
|  | ABT 6-plex | 200 | 60 | 5 | 25 |
|  | BioGerm 2-plex | 300 | / | 5 | 25 |
|  | BioPerfectus 2-plex | 140 | 60 | 5 | 25 |
|  | Daan singleplex | 200 | 100 | 5 | 25 |
| HAdV-3, HAdV-7 | Sansure 6-plex | 300 | 80 | 5 | 50 |
|  | Sansure singleplex | 300 | 80 | 10 | 50 |
|  | ABT 6-plex | 200 | 60 | 5 | 25 |
|  | BioGerm 6-plex | 300 | / | 5 | 25 |
| RSVA, RSVB | Sansure 6-plex | 300 | 80 | 5 | 50 |
|  | ABT 6-plex | 200 | 60 | 5 | 25 |
|  | BioGerm 6-plex | 300 | / | 5 | 25 |
|  | Daan singleplex | 200 | 100 | 10 | / |
| HRV-B72 | Sansure 6-plex | 300 | 80 | 5 | 50 |
|  | Daan singleplex | 200 | 100 | 10 | / |
| HPIV-1, HPIV-2, HPIV-3 | ABT 6-plex | 200 | 60 | 5 | 25 |
|  | BioGerm 6-plex | 300 | / | 5 | 25 |
|  | Hecin singleplex | 50 | 25 | 5 | 25 |
| hMPV-A2 | BioGerm 6-plex | 300 | / | 5 | 25 |
|  | Daan singleplex | 200 | 100 | 5 | 25 |

Abbreviations: Omicron BA.5, severe acute respiratory syndrome coronavirus 2 Omicron BA.5 strain; H1N1pdm09, pandemic influenza A (H1N1) 2009 virus; H3N2, influenza A (H3N2) virus; B/Victoria, Victoria-lineage of influenza B virus; HAdV-3, HAdV-7, human adenovirus type 3, 7; RSVA, RSVB, respiratory syncytial virus subtypes A, B; PIV-1, -2, -3, parainfluenza virus type 1, 2, 3; hMPV-A2, human metapneumovirus A2 strain; HRV-B72, human rhinovirus B72 strain.

**REFERENCES**

1. Maertzdorf J, Wang CK, Brown JB, Quinto JD, Chu M, de Graaf M, van den Hoogen BG, Spaete R, Osterhaus AD, Fouchier RA. 2004. Real-time reverse transcriptase PCR assay for detection of human metapneumoviruses from all known genetic lineages. J Clin Microbiol 42:981-6.

2. U.S. Food and Drug Administration. Available from: <https://www.accessdata.fda.gov/scripts/cdrh/cfdocs/cfPMN/pmn.cfm?ID=K200370>. Accessed 5 June 2024.

3. World Health Organization. WHO information for molecular diagnosis of influenza virus - updateinformation for molecular diagnosis of influenza virus - update. Available from: <https://www.who.int/teams/global-influenza-programme/laboratory-network/quality-assurance/eqa-project/information-for-molecular-diagnosis-of-influenza-virus>. Accessed 5 June 2024.

4. NATIONAL INSTITUTE FOR VIRAL DISEASE CONTROL AND PREVENTION, CHINA CDC. Specific primers and probes for detection 2019 novel coronavirus. Available from: <https://ivdc.chinacdc.cn/kyjz/202001/t20200121_211337.html>. Accessed 5 June 2024.

5. Hu A, Colella M, Tam JS, Rappaport R, Cheng SM. 2003. Simultaneous detection, subgrouping, and quantitation of respiratory syncytial virus A and B by real-time PCR. J Clin Microbiol 41:149-54.

6. Heim A, Ebnet C, Harste G, Pring-Akerblom P. 2003. Rapid and quantitative detection of human adenovirus DNA by real-time PCR. J Med Virol 70:228-39.

7. van de Pol AC, van Loon AM, Wolfs TF, Jansen NJ, Nijhuis M, Breteler EK, Schuurman R, Rossen JW. 2007. Increased detection of respiratory syncytial virus, influenza viruses, parainfluenza viruses, and adenoviruses with real-time PCR in samples from patients with respiratory symptoms. J Clin Microbiol 45:2260-2.

8. Lu X, Holloway B, Dare RK, Kuypers J, Yagi S, Williams JV, Hall CB, Erdman DD. 2008. Real-time reverse transcription-PCR assay for comprehensive detection of human rhinoviruses. J Clin Microbiol 46:533-9.
